# Necrotizing Fasciitis Within 72 hours After Presentation with Skin and Skin Structure Infection

**DOI:** 10.5811/westjem.2020.5.46046

**Published:** 2020-07-10

**Authors:** Urania Rappo, H. Bryant Nguyen, Sailaja Puttagunta, Caroline Ojaimi, Karthik Akinapelli, Michael W. Dunne

**Affiliations:** * Allergan PLC, Madison, New Jersey; † Loma Linda University, Division of Pulmonary, Critical Care, Hyperbaric, Allergy and Sleep Medicine Loma Linda, California; ‡ Independent consultant, Hamden, Connecticut; § BiomX Ltd, Ness Ziona, Israel; ¶ Novartis Pharmaceuticals Corp, East Hanover, New Jersey; || Iterum Therapeutics, Old Saybrook, Connecticut

## Abstract

**Introduction:**

A small percentage of patients with skin infections later develop necrotizing fasciitis (NF). Diagnostic testing is needed to identify patients with skin infections at low risk of NF who could be discharged from the emergency department (ED) after antibiotic initiation. Elevated lactate has been associated with NF; existing estimates of the frequency of NF are based on retrospective reviews, and cases often lack testing for lactate. We present the incidence of patients with skin infections who developed NF and their baseline lactates.

**Methods:**

In four phase-3 trials, 2883 adults with complicated or acute bacterial skin and skin structure infections were randomized to dalbavancin or comparator, with early and late follow-up visits through Day 28. We prospectively collected baseline plasma lactates in one trial to assess an association with NF.

**Results:**

NF was diagnosed in 3/2883 patients (0.1%); all three survived. In the study with prospectively collected baseline lactates (n = 622), 15/622 (2.4%) had a lactate ≥4 millimoles per liter (mmol/L), including 3/622 (0.5%) with a lactate ≥7 mmol/L. NF was not seen in patients with a lactate <4 mmol/L; NF was seen in 1/15 (6.7%) with a lactate ≥4 mmol/L, including 1/3 (33.3%) with lactate ≥7 mmol/L.

**Conclusions:**

NF incidence within 72 hours of antibiotic initiation in patients with complicated or acute bacterial skin and skin structure infections was extremely low (0.1%) and occurred in 6.7% with a lactate ≥4 mmol/L. Lactate <4 mmol/L can be used to identify patients at low risk of NF who could be safely discharged from the ED after antibiotic initiation.

## INTRODUCTION

A small percentage of patients with serious skin infections later develop life-threatening necrotizing fasciitis (NF). NF has an annual incidence ranging from 0.3–15.5 cases per 100,000 population.^1^ It involves the epidermis, dermis, subcutaneous tissue, fascia, and muscle, and is a surgical diagnosis, characterized by friable superficial fascia and dishwater-gray exudate.^1^ NF can occur after minor or major breaches in skin or mucosa and requires emergent and extensive surgical debridement.^1^ It is defined as polymicrobial (type I) involving aerobic and anaerobic organisms with gas in the tissue in patients with underlying conditions such as diabetes, while monomicrobial NF (type II) most commonly involves *Streptococcus pyogenes*, followed by methicillin-resistant *Staphylococcus aureus* (MRSA), and can occur in persons without underlying conditions.^1^ In invasive skin infections caused by *S. pyogenes*, the initial lesion may be mildly erythematous and swollen but progress to extensive inflammation over the next 24 to 72 hours with the skin appearing as dusky, then purplish with bullae, crepitus and/or subcutaneous gas, and with an associated mortality of 30–80%.^1^–^3^

Since the initial clinical presentation may be benign and not suggestive of NF, a common clinical question in the emergency department (ED) is whether patients with skin infection can be discharged after antibiotic initiation, or if they may be at higher risk of NF and therefore require a brief hospital admission for observation while receiving intravenous (IV) antibiotic therapy. Adjunctive diagnostic testing is therefore needed to triage patients with skin infections at low risk of NF, who could potentially be safely discharged from the ED. Elevated lactate has been associated with NF and mortality (lactate level >2 or ≥6 millimoles per liter [mmol/L]),^4^,^5^ mortality in patients with infection (lactate level ≥4 mmol/L),^6^ and septic shock (lactate level >2 mmol/L).^7^ Existing estimates of the frequency of NF are based on retrospective reviews, and cases often lack prospective testing for lactate.

Dalbavancin is a long-acting lipoglycopeptide antibiotic approved by the US Food and Drug Administration and European Medicines Agency as a single- and two-dose treatment for adults with acute bacterial skin and skin structure infections (ABSSSI) caused by susceptible Gram-positive organisms, including MRSA.^8^–^10^ Dalbavancin has a terminal half-life of 15.5 days and is administered as a single IV infusion in the inpatient or outpatient setting to provide a complete two-week course of therapy for ABSSSI, eliminating the need for a peripherally inserted central catheter. Clinicians may be concerned about the risk of NF developing after patients present with skin infection in the ED, and many patients may be admitted unnecessarily to the hospital for IV antibiotics and observation.

In this analysis, we present the incidence of patients initially presenting with skin and skin structure infections (SSSI) who later developed NF, with available baseline lactate levels from four global phase-3 clinical trials of dalbavancin in SSSI, including 386 patients treated completely in the outpatient setting.^11^

## METHODS

This analysis evaluated data from four phase-3, double-blind, placebo-controlled multicenter trials of 2883 adults with complicated (cSSSI) or ABSSSI. Detailed methods for those trials have been described previously; patients were randomized to receive dalbavancin or linezolid (VER001-9, no registry number – study was completed prior to establishment of Clinicaltrials.gov),^8^ dalbavancin or vancomycin (with oral switch to linezolid) (DUR001-301, NCT01339091, and DUR001-302, NCT01431339)^10^ or dalbavancin single-dose or two-dose regimen (DUR001-303, NCT02127970).^9^ The study protocols included multiple early and late follow-up visits through Day 28. Based on an observation of two prior cases in studies VER001-9 and DUR001-302 of patients who presented with symptoms of cSSSI or ABSSSI and later developed NF, including one from DUR001-302 with an elevated baseline lactate level (4.4 mmol/L), the most recent study, DUR001-303, was designed to prospectively collect baseline plasma lactate levels as an exploratory analysis to assess an association with NF in all patients presenting with SSSI. The protocols for all studies were approved by the institutional review board or ethics committee at each study site, and all patients provided written informed consent.

**Population Health Research Capsule**

What do we already know about this issue?

*A simple diagnostic tool is needed to triage patients with skin infection at low risk of necrotizing fasciitis (NF), who could be discharged from the emergency department (ED) after antibiotics*.

What was the research question?

*We prospectively collected baseline lactates in patients presenting with skin infections, to assess an association with NF*.

What was the major finding of the study?

*Lactate <4 millimoles per liter can identify patients at low risk of NF, who could be safely discharged from the ED after antibiotic initiation*.

How does this improve population health?

*A lactate level in the ED could identify patients at low risk of NF, to avoid unnecessary hospital admissions*.

### Key Inclusion Criteria

Patients ≥18 years of age with cSSSI or ABSSSI involving deeper soft tissue or needing significant surgical intervention (major cutaneous abscess *or* surgical site or traumatic wound infection *or* cellulitis) were enrolled into the trials. Patients must have presented with at least one of the following systemic signs of infection: an elevated body temperature *or* increased white blood cell count *or* white blood differential count with ≥10% band forms. In addition to erythema, at least two of the following signs of ABSSSI were required: purulent drainage/discharge; fluctuance; heat/localized warmth; tenderness to palpation; or swelling/induration.

### Key Exclusion Criteria

We excluded patients if they had evidence of NF at enrollment, gas gangrene, or gangrene, or if infections were expected to require more than two surgical interventions for the cSSSI or ABSSSI. Patients were also excluded from studies DUR001-301, -302, and -303 if they had sustained shock at enrollment, defined as systolic blood pressure <90 millimeters mercury for >2 hours despite adequate fluid resuscitation, with evidence of hypoperfusion or need for sympathomimetic agents to maintain blood pressure.

### Study Outcomes

Patients were selected for this analysis from the four trials if they had baseline lactate levels available (DUR001-302 and DUR001-303) and/or if they developed NF. If signs or symptoms of NF were observed at the prior site of skin and soft tissue infection after enrollment, the treating physicians and surgical team established the diagnosis by surgical exploration in the operating room.

### Statistical Analysis

We used sensitivity, specificity, positive predictive value, and negative predictive value to determine the predictive value of lactate in determining NF.^12^,^13^ Confidence intervals (CI) for sensitivity and specificity are “exact” Clopper-Pearson CIs;^12^ CIs for positive predictive value and negative predictive value are the standard logit CIs.^13^

## RESULTS

**Figure 1:**
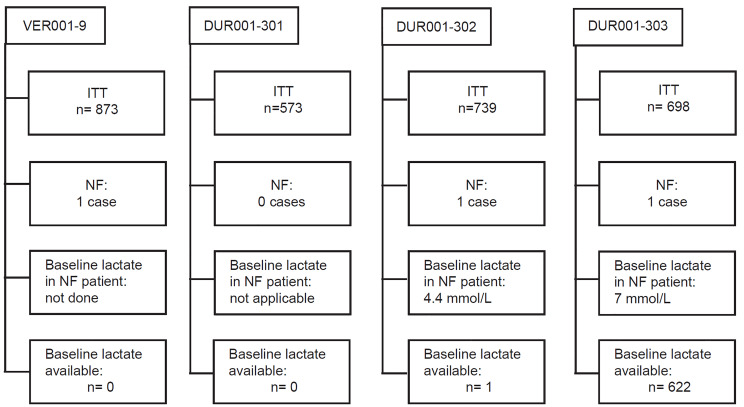
Incidence of necrotizing fasciitis and baseline lactate levels in intent-to-treat population. *ITT*, intent-to-treat; *NF*, necrotizing fasciitis; *mmol/L*, millimoles per liter.

**Figure 2:**
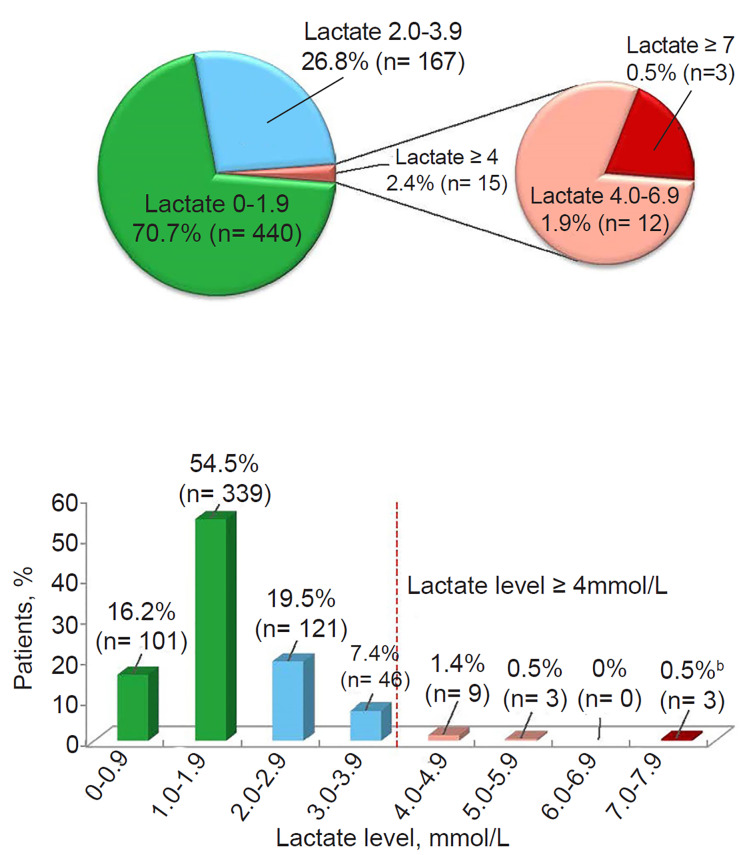
Baseline lactate levels in DUR001-303 (N = 622).^a^ ^a^622 ITT patients had available baseline plasma lactate levels (normal range, 0.5–2.2 mmol/L). Figure includes 20 patients with lactate = 2.0 mmol/L. ^b^Case of necrotizing fasciitis in DUR001-303 (lactate = 7 mmol/L). *ITT*, intention-to-treat; *mmol/L*, millimoles per liter.

NF was diagnosed in 3/2883 patients (0.1%) within 72 hours of presentation for skin infection; all three had surgical debridement and survived (Figure 1). Clinical features of the patients who developed NF are presented in Table 1. None of these patients had evidence of NF or hemodynamic compromise consistent with septic shock at enrollment. In the DUR001-303 study, 622 patients had prospectively collected baseline plasma lactate levels per protocol: 15/622 patients (2.4%) had a lactate level ≥4 mmol/L, including 3/622 patients (0.5%) with a lactate level ≥7 mmol/L, one of whom developed NF (Figure 2). In patients with lactate levels >2 mmol/L (162/622 [26.0%]), there was no evidence of septic shock at enrollment. NF was not seen in patients with a lactate level <4 mmol/L. One of 15 patients (6.7%) with a lactate level ≥4 mmol/L had NF (lactate = 7 mmol/L); this patient constituted 1/3 patients (33.3%) with a lactate level ≥7 mmol/L. A lactate cutoff of ≥4 mmol/L in the DUR001-303 study provides a sensitivity of 100%, specificity of 97.8%, positive predictive value of 6.7%, and negative predictive value of 100% for NF (Table 2). In the earlier study (DUR001-302), the patient who developed NF had a serum sample collected at baseline that was later tested for lactate and was found to be 4.4 mmol/L (Table 1). Among the three patients with NF across the four clinical trials, 2/3 (67%) had type 1 diabetes, and 2/3 (67%) had *S. pyogenes* isolated from intraoperative specimens at the site of infection.

## DISCUSSION

Our findings suggest that initial lactate levels <4 mmol/L may identify SSSI patients at low risk of NF. The high negative predictive value of 100% for a lactate <4 mmol/L may be relevant for ruling out NF, since no patient from our studies with available lactate levels developed NF with a baseline lactate <4 mmol/L. Our results also support an association between elevated lactate levels and NF, as previously reported.^4^,^5^ One existing tool to assist in the earlier diagnosis of NF is the Laboratory Risk Indicator for Necrotizing Fasciitis (LRINEC) score, which uses six serum values to distinguish between soft-tissue infections and NF: C-reactive protein, total white blood cell count, hemoglobin, sodium, creatinine, and glucose.^14^ A LRINEC score of ≥6 in adults should raise the suspicion of NF, while a score of ≥8 is strongly predictive.^14^ The positive predictive value of a LRINEC score of ≥5.8 for NF ranged from 57–92% in three studies, with negative predictive values of 86% and 96% in two studies.^1^ In our studies, two of the three NF patients had all six serum values available, with LRINEC scores of 6 and 7, and corresponding lactate levels of 4.4 and 7.0 mmol/L, respectively. Using an initial lactate level to help rule out NF in patients presenting with SSSI may be easier than a scoring system requiring six serum values and may also provide a higher negative predictive value.

## LIMITATIONS

A limitation of this analysis is that lactate levels were not available in all 2883 patients enrolled in the four trials; rather, they were available in patients enrolled in the most recent clinical trial, DUR001-303, where baseline lactates were required by the protocol. This limitation may have been unavoidable as the DUR001-303 protocol was designed to collect baseline lactates in all patients after the observation of a high baseline lactate in the NF patient from a prior study (DUR001-302) who had a serum sample retrospectively tested with value of 4.4 mmol/L. The number of baseline samples tested in the most recent study (n = 622) is robust and allows the calculation of a meaningful sensitivity (100%), specificity (97.8%), and negative predictive value (100%) for lactate as a predictor of NF; the low prevalence of NF may be a limitation in the interpretation of NPV and PPV. Due to the small number of cases of NF, it may not be possible to draw definite conclusions, and larger analyses with more cases of NF may be useful to confirm the association.

## CONCLUSION

Overall, the incidence of necrotizing fasciitis within 72 hours of antibiotic initiation in cSSSI or ABSSSI patients was extremely low (0.1%). Patients with a lactate ≥4 mmol/L may be considered at significantly higher risk for NF. Lactate levels <4 mmol/L may identify cSSSI or ABSSSI patients at low risk of NF, who could be safely discharged from the ED after antibiotic initiation with careful follow-up. Further investigation of initial lactate levels as a predictor of NF risk are needed in patients presenting with SSSI.

## Figures and Tables

**Table 1:** Characteristics of individual patients who developed necrotizing fasciitis.

Variable	VER001-9	DUR001-302	DUR001-303
Age (years)	37	32	31
Gender	Male	Female	Male
Race/ethnicity	Asian	Other^a^	White
Infection type	Abscess	Cellulitis/Erysipelas	Cellulitis
Location of infection	Right forearm	Right arm and hand	Left buttock
C-reactive protein, mg/L	ND	>300^b^	>300^c^
WBC count, cells/mm^3^	35.3	19.1	22.2
Hemoglobin level, g/dL	15.6	13.5	11.9
Sodium level, mmol/L	127	138	135
Creatinine level, mg/dL	1.3	1.22	0.78
Glucose level, mg/dL	538	97	252
Lactate (mmol/L)	ND	4.4	7
SIRS criteria	Yes	Yes	Yes
Temperature	38.4°C	38.1°C	38.2°C
Baseline pain score	Moderate	10/10	5/10
Pathogen (infection site)	MRSA	*Streptococcus pyogenes*	*Streptococcus agalactiae*
	*Streptococcus pyogenes* (baseline wound culture)	(Day 2 intraoperative specimen from fasciotomy for NF)	(growth from baseline skin culture and intraoperative specimen during debridement on Day 4)
Clinical course prompting OR evaluation	Severe swelling, erythema and tenderness on Day 1	Worsening cellulitis on Day 2 with increased lesion area, severe pain, edema, hyperemia, a necrotic area with hemorrhagic border, new fluctuance, elbow in forced flexion, a 10 cm purulent, denuded area surrounded by hyperemia, with symptoms of fever, chills and nausea.	Worsening cellulitis 67 hours after study drug initiation, spread of infection from left buttock to left upper leg with injury of the fascia, severe fluctuance, and purulent drainage on dressing changes.
Intraoperative findings at diagnosis of NF	Surgical incision and drainage and debridement revealed copious purulence and significant necrosis of SQ tissues down to major fascia investing muscle bundles; NF diagnosed on Day 4. Required wound vac dressing, wet-to-dry dressing changes, and skin graft.	Upper extremity SQ fasciotomy; NF diagnosed on Day 2. Required additional debridements, wound revision, and skin graft.	Surgical incision and drainage and wide surgical debridement of necrotic tissue revealed putrid liquefaction and necrosis of SQ fat and fascia; NF diagnosed on Day 4.
Baseline blood culture	No growth	No growth	*Micrococcus luteus, Cutibacterium acnes*
Randomization arm	Linezolid	Dalbavancin (two-dose)	Dalbavancin (two-dose)
Infection area at baseline	375 cm^2^	1452 cm^2^	1139.7 cm^2^
Medical history	Type 1 diabetes Hypertension Hyperlipidemia	Right hand eczema Deep venous thrombosis Demyelinating process of right elbow	Type 1 diabetes Pancreatic necrosis

a Other race as noted in clinical study report: Gypsy;

b LRINEC score = 6;

c LRINEC score = 7.

*LRINEC*, Laboratory Risk Indicator for Necrotizing Fasciitis; *MRSA*, methicillin-resistant Staphylococcus aureus; *ND*, not done; *NF*, necrotizing fasciitis; *cm*, centimeter; *vac*, vacuum-assisted closure; *OR*, operating room; *SIRS*, systemic inflammatory response syndrome; *SQ*, subcutaneous; *WBC*, white blood cell.

**Table 2:** Evaluation of baseline lactate Level ≥4 millimoles per liter as predictor of necrotizing fasciitis.^12^

Test characteristic	Outcome, % (95% CI)
Sensitivity	100 (2.5–100)
Specificity	97.8 (96.3–98.8)
Positive predictive value	6.7 (4.1–10.7)
Negative predictive value	100
